# Efficacy and safety of rechallenge therapy with [177Lu]Lu-PSMA in metastatic castration-resistant prostate cancer: a systematic review and meta-analysis

**DOI:** 10.1007/s00259-025-07438-1

**Published:** 2025-07-24

**Authors:** Zineddine Belabaci, Leonie Schmidt, Mouhammed Sleiay, Felipe Couñago, Fernando López Campos, Marwan Tolba, Thomas Zilli, Ali Afshar-Oromieh, Mohamed Shelan

**Affiliations:** 1 Faculty of Medicine, Djillali Liabes University, Sidi Bel Abbes, Algeria; 2 Department of Radiation Oncology, Inselspital Bern, University of Bern, Bern, Switzerland; 3 Faculty of Medicine, Hama University, Hama, Syria; 4 Department of Radiation Oncology, University Hospital Quironsalud, Universidad Europea de Madrid, Madrid, Spain; 5 Department of Radiation Oncology, Hospital Universitario Ramón y Cajal, Madrid, Spain; 6 Department of Radiation Oncology, Dalhousie University, Halifax, Canada; 7 Department of Radiation Oncology, Oncology Institute of Southern Switzerland, EOC, Bellinzona, Switzerland; 8 Faculty of Biomedical Sciences, Università Della Svizzera Italiana, Lugano, Switzerland; 9 Faculty of Medicine, University of Geneva, Geneva, Switzerland; 10 Department of Nuclear Medicine, Bern University Hospital, Inselspital, University of Bern, Bern, Switzerland

**Keywords:** Prostate cancer, Radioligand therapy, Prostate-specific membrane antigen, [¹⁷⁷Lu]Lu-PSMA, [²²⁵Ac]Ac-PSMA, Rechallenge

## Abstract

**Background:**

Lutetium-177 PSMA radioligand therapy ([¹⁷⁷Lu]Lu-PSMA-RLT) is an effective treatment option for patients with metastatic castration-resistant prostate cancer (mCRPC). Prospective studies reported favourable efficacy and safety outcomes of up to 6 cycles of [¹⁷⁷Lu]Lu-PSMA. This study aimed to evaluate the efficacy and safety of [¹⁷⁷Lu]Lu-PSMA rechallenge therapy in patients with mCRPC who progressed after an initial course of [¹⁷⁷Lu]Lu-PSMA-RLT.

**Methods:**

This systematic review followed the Preferred Reporting Items for Systematic Reviews and Meta-analyses (PRISMA) guidelines. A systematic search was performed using relevant keywords in PubMed, EMBASE, and Scopus from establishment to March 2025. Primary endpoints included biochemical responses with a decline in prostate-specific antigen (PSA) of more than 50% and any PSA decline. Secondary outcomes included survival outcomes and treatment-related toxicity following rechallenge therapy with [¹⁷⁷Lu]Lu-PSMA. A random-effects model was used to generate pooled proportions through meta-analysis.

**Results:**

Eleven studies with 307 patients were included in the final analysis. Of these, 196 received 177Lu-PSMA RLT alone, and 111 received tandem 177Lu/225Ac-PSMA RLT. The pooled proportions of patients with more than a 50% PSA decline and any PSA decline were 0.45 (95% CI: 0.36–0.54) and 0.71 (95% CI: 0.61–0.80), respectively. In a total of 102 patients, 44 (43%) showed low-grade 1–2 xerostomia; however, no cases of serious xerostomia (grade ≥ 3) were reported. Moreover, the pooled proportion of patients experiencing grade ≥ 3 toxicity was 0.14 (95% CI: 0.09–0.19).

**Conclusion:**

Rechallenge therapy with [¹⁷⁷Lu]Lu-PSMA is a feasible and safe treatment option for late/end mCRPC patients. Tandem approaches with [225Ac]Ac-PSMA may help expand understanding of how to optimize outcomes after [¹⁷⁷Lu]Lu-PSMA progression. However, these findings require confirmation in prospective, randomized studies comparing different rechallenge strategies to define optimal sequencing and patient selection criteria in advanced prostate cancer.

## Introduction

Prostate cancer is the second most commonly diagnosed cancer and a leading cause of cancer-related death among men worldwide [1]. For patients with hormone-sensitive disease, the standard of care involves androgen deprivation therapy (ADT), now frequently combined with docetaxel or androgen receptor pathway inhibitors (ARPIs) to improve outcomes in the metastatic setting [2, 3]. However, progression despite castrate levels of serum testosterone (< 50 ng/dL) defines the transition to metastatic castration-resistant prostate cancer (mCRPC), a stage associated with poor prognosis and limited survival [2].

For certain patients, treatment options for metastatic castration-resistant prostate cancer (mCRPC) include PARP inhibitors, ARPI, radium-223, taxane chemotherapies, or the combination of any of these therapeutic groups [4, 5]. Although these therapies have demonstrated survival benefits, the median extension in overall survival (OS) per treatment line is typically limited to 3–4 months [6, 7]. As a result, there is an ongoing need for additional therapeutic strategies to manage this progressive and ultimately fatal disease.

Targeted radionuclide therapy (TRT) against prostate-specific membrane antigen (PSMA) has been considered a potential experimental treatment for mCRPC throughout the past 10 years. Specifically, the Lu-PSMA, a beta-emitter having a maximum path length of 1.8 mm and a mean of 0.7 mm [8], resulting in promising efficacy and safety outcomes after prospective clinical trials, so it got approved by the Food and Drug Administration (FDA) and the European Medicines Agency (EMA) for mCRPC patients who had previously had at least one course of taxane-based chemotherapy and ARPI [9, 10]. Lu radionuclide was selected to conjugate PSMA because of its half-life, cost-effectiveness, and emission type (beta particles) [11]. Despite these advances, a substantial proportion of patients either do not respond to Lutetium-177 PSMA (177Lu-PSMA) or experience disease progression after an initial response. Patients have very few alternatives for treatment at this relatively advanced stage of the disease. It may make sense to conduct a PSMA radioligand therapy (PSMA-RLT) again, especially for patients who have previously responded well to the therapy strategy. However, evidence supporting the efficacy and safety of such an approach remains limited, and existing data are fragmented across small, heterogeneous studies. This systematic review and meta-analysis aims to synthesize available evidence on the efficacy and safety of [¹⁷⁷Lu]Lu-PSMA rechallenge therapy in patients with mCRPC who progressed after an initial course of this PSMA-RLT. By standardizing outcomes such as overall survival, prostate-specific antigen (PSA) response, and treatment-related toxicity, this study seeks to provide a clearer understanding of the potential role of PSMA-RLT rechallenge in clinical practice.

## Methods

The Preferred Reporting Items for Systematic Reviews and Meta-analyses (PRISMA) guidelines were followed in preparing and writing this meta-analysis [12]. We registered this review in the International Prospective Register Of Systematic Reviews (PROSPERO) under No. CRD42025641589.

### Search strategy and study selection

Using the biomedical databases PubMed/MEDLINE, EMBASE, and Scopus, we performed an extensive literature search in January 2025 to find English-language publications published within the previous 7 years. We searched across the literature for studies concerning rechallenge therapy with [¹⁷⁷Lu]Lu-PSMA treatment in late-stage prostate cancer after progression on the first [¹⁷⁷Lu] Lu-PSMA treatment course using the following terms: PSMA, Lu-PSMA, prostate-specific membrane antigen, metastatic castration-resistant prostate cancer, Lutetium, Lu, or Lu-177. Published search hedges were used to limit the search results to consensus statements, retrospective and prospective cohort studies, and randomized clinical trials (RCTs). Two reviewers independently reviewed the abstracts and titles in the first step. Following a full-text review, manuscripts were sorted by section and evaluated for inclusion eligibility. Any disagreement was solved by discussion or adding a third reviewer if it’s necessary. We included the studies in the meta-analysis that met the following inclusion criteria: Adult men (age > 18 years) diagnosed with mCRPC; biochemical response to the initial [¹⁷⁷Lu]Lu-PSMA course; [¹⁷⁷Lu]Lu-PSMA rechallenge therapy following a progression-free time period and renewed progression, or insufficient response. Exclusion criteria were: basic science studies, non-human research, guidelines, editorials, case reports, narrative reviews, commentary, non-English articles, studies on non-mCRPC populations (including metastatic castration-sensitive prostate cancer), and reports limited to biodistribution or dosimetry.

### Data collection and evaluation process

We extracted the following data from individual studies: authors, study year, study design, country, patients' age, PSA, sample size, duration of therapy break before rechallenge sequences, the extent percentage of metastasis, initial Lu-PSMA agent, rechallenge agent, number of cycles, activity of rechallenge GBq/cycle. We obtained the information directly from the papers. Certain studies failed to provide the exact number for the median cycles of therapy; instead, they reported the number of cycles for each patient. Primary efficacy outcomes were: (1) any PSA decline; (2) PSA decline ≥ 50%; and (3) median overall survival (OS) with 95% confidence intervals (CIs). PSA progression-free survival (PSA-PFS) was also evaluated where available. Safety outcomes included the incidence of grade 3–5 adverse events, with a focus on anemia, leukopenia, thrombocytopenia, nephrotoxicity, and xerostomia, graded according to the Common Terminology Criteria for Adverse Events (CTCAE).

### Quality assessment

We used the Newcastle–Ottawa scale (NOS), a scoring approach to non-randomized studies in meta-analysis, to evaluate the quality of the individual studies [13]. The scale comprises 3 items: selection, comparability, and outcome. The scores for the three groups are calculated using a star system. A total score of ≥ 7 was considered good quality, and a total score of < 5 was considered poor quality.

### Statistical analysis

Statistical analyses were performed utilizing STATA Version 17. The effect size was calculated as an event rate accompanied by a 95% confidence interval (CI). We reported pooled proportions employing a random-effects model. Forest plots were generated to illustrate the biochemical responses, specifically addressing any reduction in PSA levels, as well as a PSA reduction of 50%, alongside grade 3–5 adverse events. The heterogeneity among individual studies was assessed using Cochran's Q and the I^2^ statistic. I^2^ values of 25%-49%, 50%-74%, and 75% or higher were classified as indicative of mild, moderate, and high heterogeneity, respectively. The Egger test and visual assessment of funnel plots were employed to evaluate potential publication bias. P-values below 0.05 were deemed statistically significant.

## Results

### Study selection

**Fig. 1:**
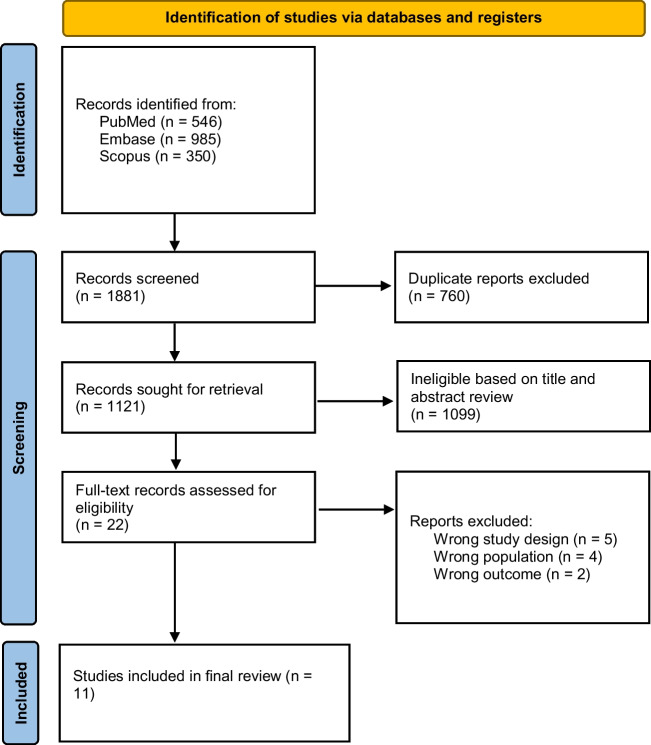
PRISMA flow diagram

The initial database search yielded 1,881 records. After removing 760 duplicates, 1,121 articles were screened by title and abstract. Of these, 22 were selected for full-text review, and 11 studies [14–24] met our criteria and were included in our meta-analysis. Ten were observational studies, and one was a single-arm phase II prospective trial. The PRISMA flow diagram illustrates the study selection process (Fig. 1).

### Study characteristics

A total of 307 patients were included in our meta-analysis, which consisted of 7 articles with 196 patients receiving 177Lu-PSMA RLT and 4 articles with 111 patients undergoing a tandem therapy of 177Lu-PSMA RLT and 225Ac-PSMA RLT. The median PSA value before [¹⁷⁷Lu]Lu-PSMA rechallenge therapy ranged from 52 to 215 ng/mL. The number of cycles for the rechallenge course varied from 1 to 7, with an average of 3 cycles, and studies reported varying administered activity per cycle. The percentage of affected sites indicated the extent of metastasis: bone, lymph nodes, and visceral organs. Detailed study characteristics are summarized in (Table 1).

**Table 1:** Overview of studies characteristics

Study	Study design (R/P), country	Initial Agent	Rechallenge Agent	Patients (n)	Age (y)	Baseline PSA level (ng/mL)	Extent of Metastasis (%)	Duration of therapy break before rechallenge (mo, range)	Number of cycles Median (range)		Activity per cycle Median(range)	
									Initial	Rechallenge	Initial	Rechallenge
Yordanova et al. (2018) [14]	R, Germany	[177Lu]Lu-PSMA-617	[177Lu]Lu-PSMA-617	30	71.5 (51–88) mean, range	208 (2.6–2009) mean, range	Bone 97 Lymph node 87 Liver 10 Lung 13	6 ( 2–26)	3 (1–5)	3 (1–6)	Cumulative activity of 17.9 GBq	6.1 GBq (3.8–6.7)
Gafita et al. (2019) [15]	R, Germany	[177Lu]Lu-PSMA-I&T	[177Lu]Lu-PSMA-I&T	8	72 (62–77)	52 (5–2328)	Bone 87.5 Lymph node 75 Visceral 25	5.4 (3.8–14.7)	6 (4–6)	2 (1–4)	NR	NR
Kulkarni et al. (2019) [16]	Germany	[177Lu]Lu-PSMA-617	[225Ac]Ac-PSMA-617/[177Lu]Lu-PSMA-617 tandem therapy	23	NR	NR	NR	NR	NR	1–3 tandem	NR	3.5—7.5 GBq Lu-PSMA; 3—5 MBq for Ac-PSMA
Khreish et al. (2020) [17]	R, Germany	[177Lu]Lu-PSMA-617	[225Ac]Ac-PSMA-617/[177Lu]Lu-PSMA-617 tandem therapy	20	72 (57–88)	215 (6–5547)	Bone 95 Lymph node 45 Liver 20 Lung 10	NR	4 (1–13)	1 Cycle tandem; 1 (0–5) cycles of post-tandem maintenance 177Lu-PSMA-617 monotherapy	Cumulative activity of 26.3 (7.4–77.4)	6.9 (5.0–11.6) GBq Lu-PSMA; 5.3 (1.5–7.9) MBq Ac-PSMA
Violet et al. (2020) [18]	P, Australia	[177Lu]Lu-PSMA-617	[177Lu]Lu-PSMA-617	15/50 received rechallenge therapy	71 (50–87)	189.8 (7–4.022)	Bone 76 Node Only 4 Visceral 20	NR	4 (2–4)	2 (1–5)	7.5 GBq (4–8.9) (mean)	7.5 GBq (4–8.9) (mean)
Rosar et al. (2021) [19]	R, Germany	[177Lu]Lu-PSMA-617	[225Ac]Ac-PSMA-617/[177Lu]Lu-PSMA-617 tandem therapy	17	69.4 ± 8.3 (57.0–89.0)	152 ng/mL (5.9–2570 ng/mL)	Bone 100 Lymph node 29.4 Liver 11.8	NR	5 (2–9)	1 cycle tandem	Cumulative mean activity of 35 GBq (range: 14–66)	6.0 GBq (3.8–8.2) Lu-PSMA; 4 MBq (1.8–6.9) Ac-PSMA
Gäble et al. (2024) [20]	R, Germany	[177Lu]Lu-PSMA-I&T	[177Lu]Lu-rhPSMA-10.1	10	NR	108 (7.3–1700)	Bone 90 Lymph node 80 Liver 10 Lung 0	7 to 12 weeks	2–6	2 (1–3)	Cumulative activity of 14.8–44.8 GBq	7.4–8.1 GBq
Rosar et al. (2024) [21]	R, Germany	[177Lu]Lu-PSMA-617	[177Lu]Lu-PSMA-617	47	72 (58–87)	103 (1.0–5475)	Bone 91.5 Lymph node 63.8 Liver 25	After PSA-based PFS of median 10.8	3 (1–8)	2 (1–6)	6.20 GBq (4.33–9.10)	7.0 GBq (4.25–9.25)
Rosar et al. (2024) [22]	R, Germany	[177Lu]Lu-PSMA-617	[225Ac]Ac-PSMA-617/[177Lu]Lu-PSMA-617 tandem therapy	51	72.5 (51–97)	191 (6–5547)	Bone 96.1 Lymph node 66.7 Liver 15.7 Lung 7.8	NR	Mean of 4 ± 2 (2–9)	Mean of 2 ± 2 (1–8) Lu-PSMA; 2 ± 1 ( 1–6) Ac-PSMA	Mean cumulative activity of 29.9 ± 12.5 GBq (range:12.9–60.9)	Mean of 6.0 ± 1.4 GBq 177Lu; 3.9 ± 1.7 MBq 225Ac
Santo et al. (2024) [23]	R, Austria	[177Lu]Lu-PSMA-I&T	[177Lu]Lu-PSMA-I&T	18	70 (44–93)	186 ± 528	Bone 72 Lymph node 89 Visceral 22	9 (3–13)	5 (4–7)	4 (2–7)	Cumulative activity of 38.4 ± 8.76 GBq	Cumulative activity of 26.1 GBq
Seifert et al. (2024) [24]	R, Germany	[177Lu]Lu-PSMA-617 OR -I&T	[177Lu]Lu-PSMA-617 OR -I&T	68/111 received rechallenge therapy	72	NR	NR	7.2 (5.4–11.5)	6	1 to ≥ 7	6.8 GBq	6.8 GBq

Age and PSA are presented in median(range); *R* retrospective, *P* prospective

### Quality assessment

All the included observational studies showed to have a high quality after applying the NOS for the quality assessment, except one study [16] with insufficient data for full assessment, but the required outcomes of interest were reported. Moreover, using the ROBINS-I (Risk Of Bias In Non-Randomized Studies– of Interventions) assessment, the single-arm prospective trial showed low risk of bias in all domains except some concerns in the selection of participants. Further details can be found in (Table 2).

**Table 2:** Quality assessment of the included studies based on the Newcastle–Ottawa Scale

Study	Selection	Comparability	Outcome	Score
Yordanova et al. (2018) [14]	***	**	**	**7**
Gafita et al. (2019) [15]	***	*	***	**7**
Khreish et al. (2020) [17]	***	*	***	**7**
Rosar et al. (2021) [19]	***	**	***	**8**
Gäble et al. (2024) [20]	***	**	**	**7**
Rosar et al. (2024) [21]	***	**	***	**8**
Rosar et al. (2024) [22]	***	**	***	**8**
Santo et al. (2024) [23]	***	**	***	**8**
Seifert et al. (2024) [24]	***	**	***	**8**

NB:* represents one point, ** represents two points, and *** represents three points. The total score is the sum of the selection, comparability, and outcome sub-item scores

### Efficacy outcomes

#### More than 50% PSA decline

**Fig. 2:**
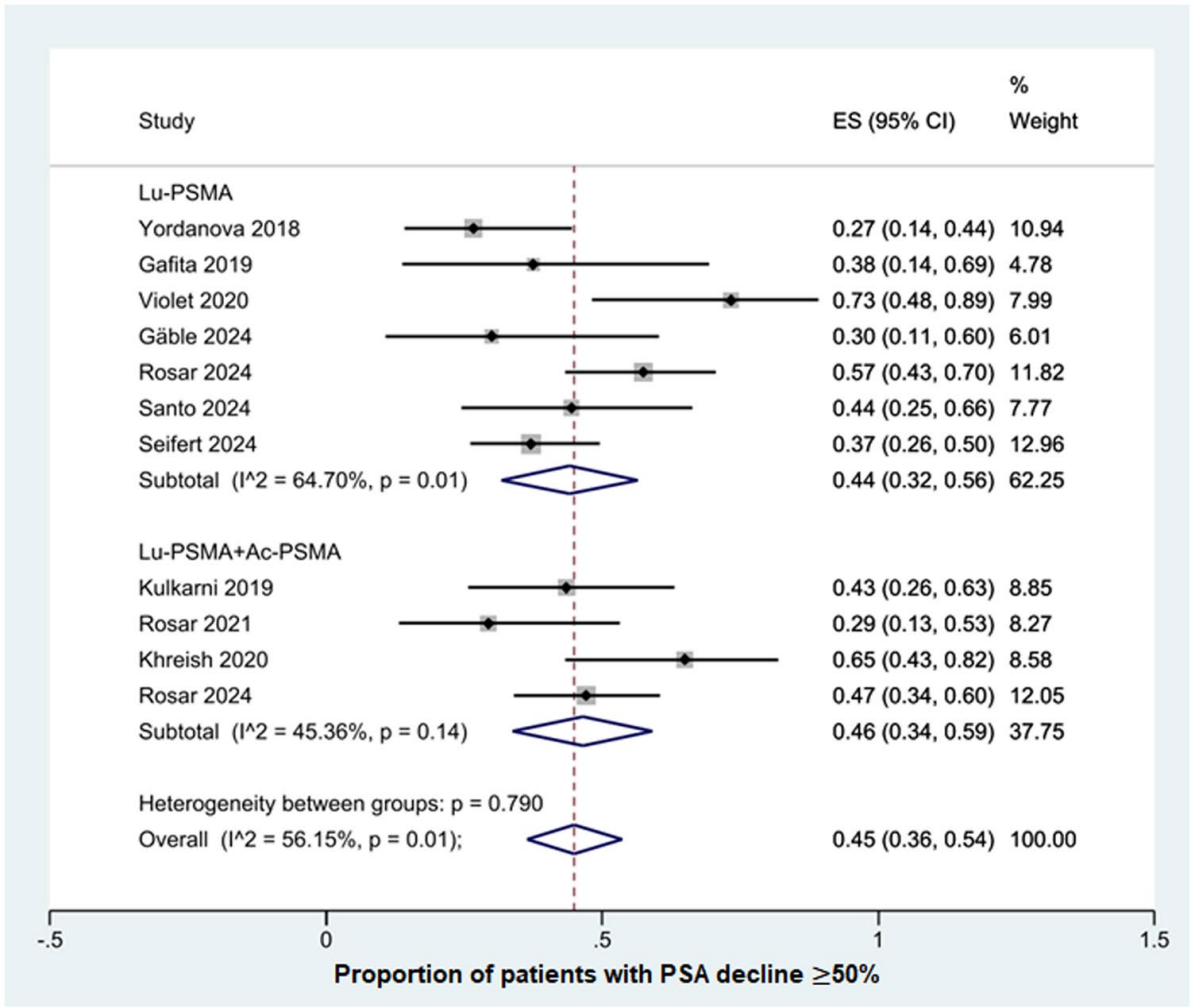
Forest plot of studies demonstrating > 50% PSA decline after rechallenge therapy

All 11 studies reported results on PSA decline of more than 50%, an overview of PSA response is shown in (Table 3). The pooled proportion of patients achieving a PSA decline ≥ 50% was 0.45 (95% CI: 0.36–0.54; Fig. 2). In the subgroup analysis, [¹⁷⁷Lu]Lu-PSMA monotherapy showed a response rate of 0.44 (95% CI: 0.32–0.56), while the combination with [²²⁵Ac]Ac-PSMA yielded 0.46 (95% CI: 0.34–0.59). Moderate heterogeneity was observed (I^2^ = 56.2%, *p* = 0.01), with no significant subgroup differences (*p* = 0.79), and the overall pooled analysis yielded a z-score of 10.23 (*p* < 0.001), confirming robust statistical significance across all studies.

**Table 3:** Overview of biochemical responses, survival and toxicity data

Study	Any PSA decline (n,( %))	PSA decline > 50% (n,( %))	PFS (mo, (95% CI))	OS (mo, (95% CI))	Grade ≥ 3 Toxicity (n,(%))				
					Anemia	Thrombocytopenia	Leukopenia	Nephrotoxicity	Xerostomia
Yordanova et al. (2018) [14]	16/27 (59)	8/30 (27)	2.8 (1–11)	12	4 (13)	4 (13)	2 (7)	1 (3)	NR
Gafita et al. (2019) [15]	6/8 (75)	3/8 (38)	3.2 (2.6–3.7)	14 (6.2–21.8)	1 (13)	2 (25)	0	0	0
Kulkarni et al. (2019) [16]	13/23 (57)	10/23 (43)	21 weeks	35 weeks	0	2 (9)	0	0	0
Khreish et al. (2020) [17]	18/20 (90)	13/20 (65)	19 (12–26) weeks	48 (4–92) weeks	3 (15)	2 (10)	2 (10)	0	0
Violet et al. (2020) [18]	NR	11/15 (73)	NR	26.6 from time of study enrollment	0	1 (7)	1 (7)	1 (7)	NR
Rosar et al. (2021) [19]	10/17 (59)	5/17 (29)	3.7 (3.0–4.4)	PR vs. SD/PD by imaging: not reached vs. 8.3 m	0	1 (6)	0	0	0
Gäble et al. (2024) [20]	5/10 (50)	3/10 (30)	NR	6 (3–14)	2 (20)	1 (10)	1 (10)	0	0
Rosar et al. (2024) [21]	40/47 (85)	27/47 (57)	8.7 (0.5–39.2)	22.7 (18.2–24.7)	6 (13)	1 (2)	3 (6)	1/47 (2)	0
Rosar et al. (2024) [22]	36/51 (71)	24/51 (47)	6.3 (3.9–10.0)	9.1 (5.4–12.8)	8 (16)	6 (12)	1 (2)	1 (2)	0
Santo et al. (2024) [23]	12/18 (67)	8/18 (44)	NR	11 (8.1–13.8)	1 (6)	1 (6)	**0**	1 (6)	**NR**
Seifert et al. (2024) [24]	NR	23/62 (37)	NR	40.2 from the first cycle of the initial course	10 (15)	3 (4)	0	5 (7)	NR

*OS* overall survival, *PFS* progression-free survival, *PR* partial response, *SD* stable disease, *PD* progressive disease, *CI* confidence interval, *NR* not reported

#### Any PSA decline

**Fig. 3:**
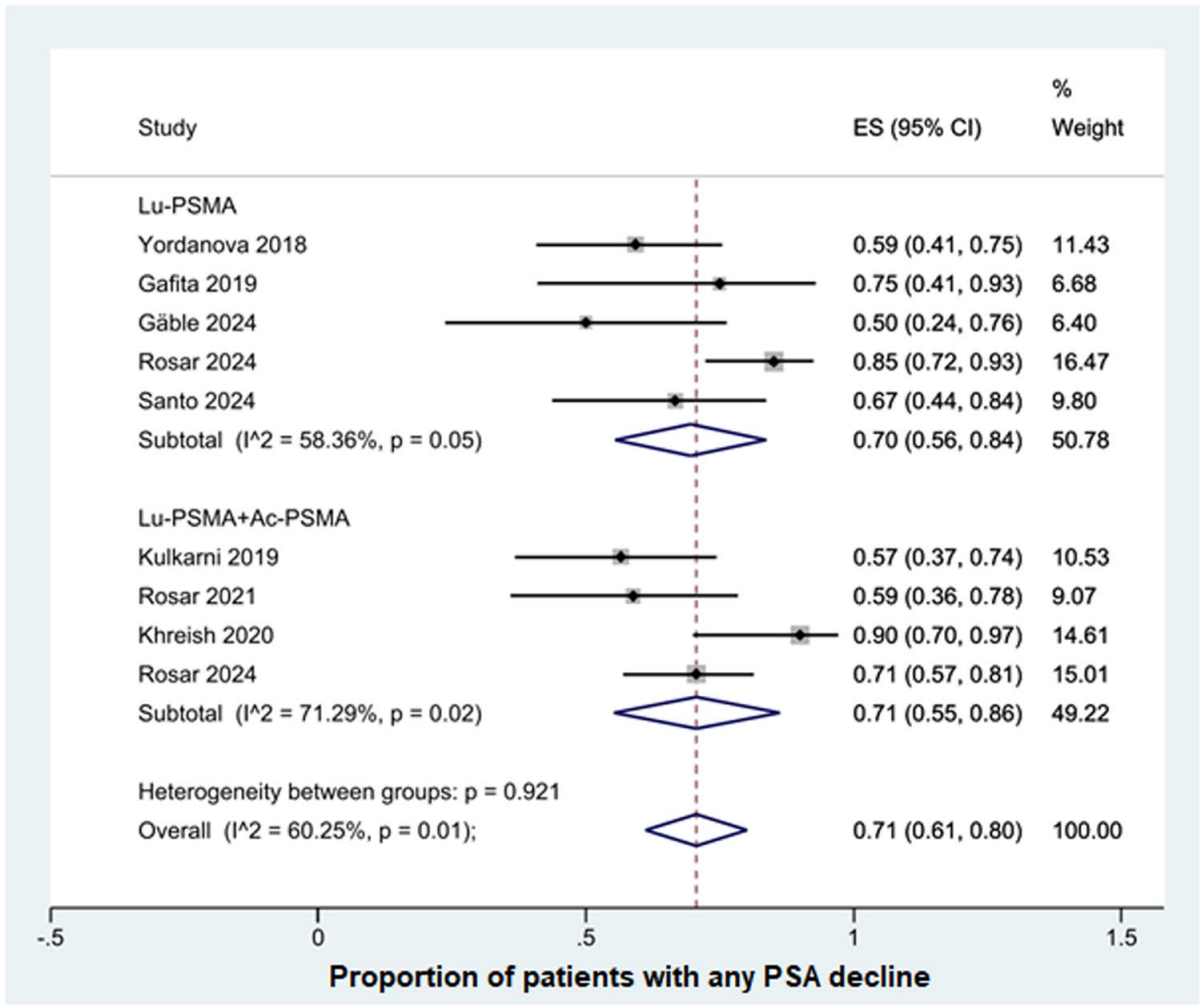
Forest plot of studies demonstrating any PSA decline after rechallenge therapy

Nine studies reported results on any PSA decline. The overall pooled proportion was 71% (95% CI, 61–80%). For the [¹⁷⁷Lu]Lu-PSMA subgroup, five studies were included, demonstrating a pooled proportion of 0.70 (95% CI: 0.56–0.84) with moderate heterogeneity (I^2^ = 58.36%, *p* = 0.05). For the 177Lu-PSMA + 225Ac-PSMA subgroup, four studies showed a pooled proportion of 0.71 (95% CI: 0.55–0.86) but higher heterogeneity (I^2^ = 71.29%, *p* = 0.02)**(**Fig. 3**)**. Both [¹⁷⁷Lu]Lu-PSMA monotherapy and in combination with [²²⁵Ac]Ac-PSMA showed similar effectiveness in reducing PSA levels during rechallenge courses, with no statistically significant difference between the two approaches.

#### Survival outcomes

All studies provided data for OS; the median OS calculated from the administration of the first rechallenge series ranged from 35 weeks to 22.7 months. The PSA-PFS was reported in seven studies, but with heterogeneous definitions. Detailed survival data are presented in (Table 3).

### Safety and adverse events

Most of the low-grade toxicity was related to the initial course of [¹⁷⁷Lu]Lu-PSMA RLT. We collected data of Grade ≥ 3 toxicity profile related to the rechallenge course in (Table 3). Ten studies provided data on grade ≥ 3 adverse events related to rechallenge therapy. The pooled proportion of patients experiencing grade ≥ 3 toxicity was 0.14 (95% CI: 0.09–0.19), with low heterogeneity (I^2^ = 12.8%, *p* = 0.33). For monotherapy, the rate was 0.17 (95% CI: 0.10–0.24); for combination therapy, 0.11 (95% CI: 0.05–0.18). No significant subgroup difference was observed (*p* = 0.244). Both therapeutic regimens were associated with statistically significant reductions in Grade 3–5 AEs during the rechallenge course, with a marginally lower proportion favoring the combination group. Five studies [15, 17, 19–21] assessed xerostomia grade 1–2 during rechallenge course. In a total of 102 patients, 44 (43%) showed low-grade 1–2 xerostomia; however, no cases of serious xerostomia (grade ≥ 3) were reported. The percentage of anemia, thrombocytopenia, and nephrotoxicity grade ≥ 3 was 11% (35/307), 7.8% (24/307), and 3.3% (10/307), respectively.

### Publication bias

**Fig. 4:**
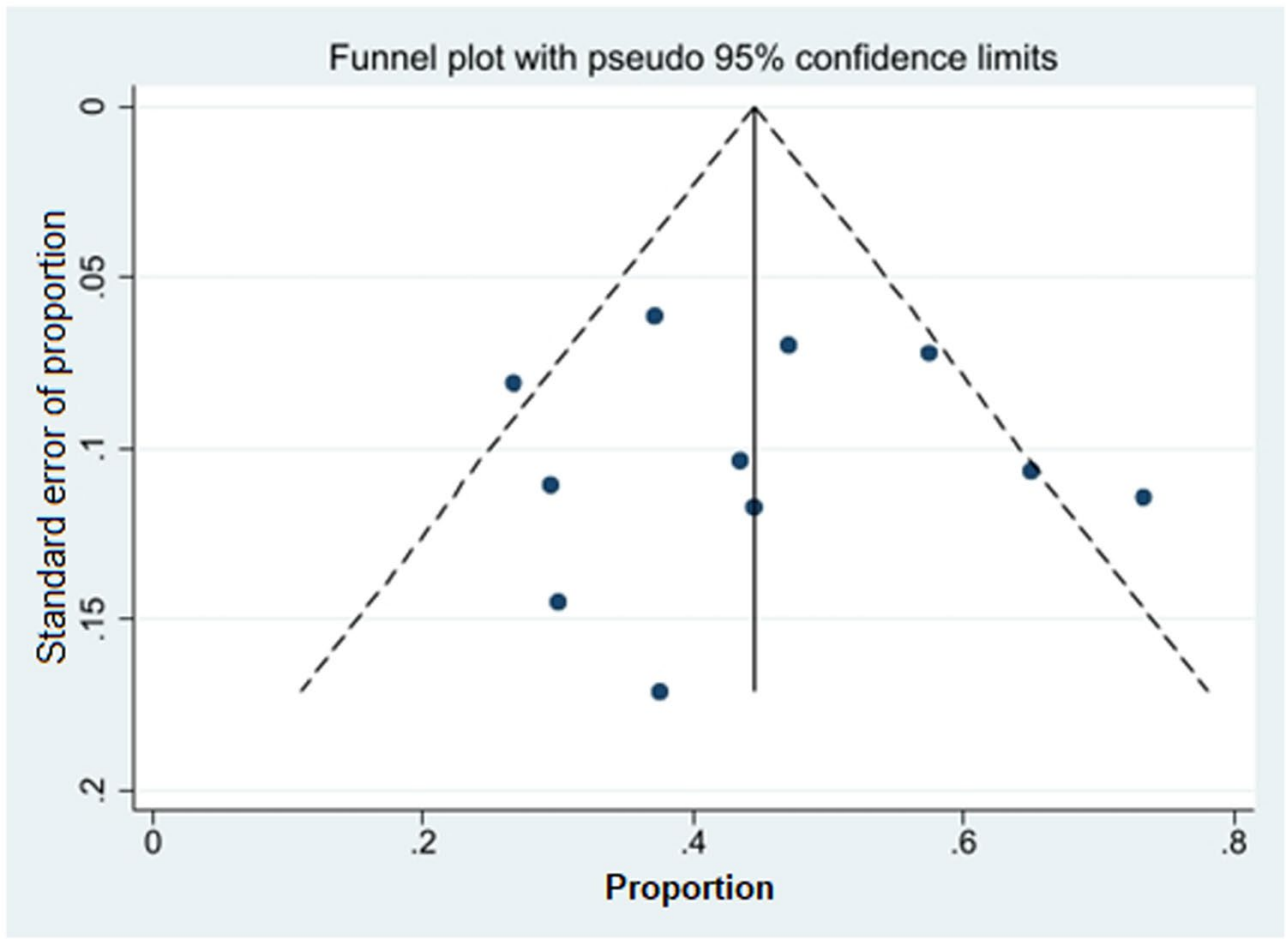
Funnel plot of publication bias for over 50% of PSA decline after rechallenge therapy

Visual inspection of the funnel plot for the PSA ≥ 50% decline outcome revealed some asymmetry, suggesting potential publication bias **(**Fig. 4**).** However, Egger’s test indicated minimal statistical evidence for such bias. Observed heterogeneity likely reflects differences in study design, sample size, and treatment regimens. The absence of significant between-group heterogeneity (*p* = 0.79) supports the validity of pooling results for an overall estimate.

## Discussion

In the clinical setting of mCRPC, there are limited therapeutic options despite advances in systemic treatments. Resistance to androgen receptor-targeted therapies and taxane-based chemotherapy remains a significant clinical challenge. Moreover, disease heterogeneity and the lack of predictive biomarkers make optimal treatment selection challenging. These challenges call for the implementation of novel and personalized treatment approaches to improve patient prognosis. This meta-analysis represents the first comprehensive and quantitatively robust synthesis of available data evaluating the efficacy and safety of [¹⁷⁷Lu]Lu-PSMA-RLT in a rechallenge setting, addressing a significant and previously underexplored gap in the management of mCRPC.

Rechallenge therapy is usually given when initial cycles show biochemical and imaging responses, but the disease progresses after PSMA-RLT cessation. Our meta-analysis demonstrates a PSA decline of ≥ 50% in 45% of patients after rechallenge—a clinically meaningful finding that underscores the retained antitumor potential of PSMA-targeted radionuclide therapy even after prior exposure. Subgroup analyses showed comparable results in both 177Lu-monotherapy (44%) and tandem therapy with ^225^Ac-PSMA (46%), confirming the consistency and robustness of the therapeutic effect across different treatment strategies. Interestingly, our results were similar to Yadav et al. [25]meta-analysis, which showed a 46% PSA 50% decline rate in mCRPC patients treated with [¹⁷⁷Lu]Lu-PSMA RLT for the first time. Same result with VISION trial (46%) [9]. However, it must be emphasized that PSMA-RLT rechallenge is typically performed in patients who previously showed a good response to the initial line of [¹⁷⁷Lu]Lu-PSMA. Consequently, the results cannot be directly compared with studies that also included patients who turned out to be non-responders to PSMA-RLT.

In our analysis, the incidence of severe adverse events appeared lower than that reported in the VISION and TheraP trials [9, 10]. This difference likely reflects not only variations in patient populations but also discrepancies in the methodology used to evaluate toxicity. Although all studies employed the CTCAE classification, differences in grading interpretation, reporting thresholds, or clinical documentation practices may have contributed to divergent safety profiles. These findings highlight the need for harmonized toxicity assessment protocols to enable more accurate cross-trial comparisons. Rathke et al. [26] investigated methods to improve the tolerability of PSMA-targeted alpha therapy (PSMA-TAT) while maintaining antitumor efficacy. One approach involved a combination of approximately 4 GBq of 177Lu-PSMA with 4 MBq of 225Ac-PSMA. This represents an approximately 50% reduction from previously recommended doses for each agent. A PSA decline greater than 50% was observed in 57% of patients, with better tolerability. These findings could support the potential of RLT combination therapy as a strategy to balance efficacy and side effects, particularly in reducing salivary gland toxicity. This becomes increasingly relevant the earlier PSMA-RLT is implemented in the treatment of prostate cancer.

Similarly, promising survival outcomes were observed, Rosar et al. [21], analyzing data from the prospective registry (REALITY Study), reported a median OS of 22.7 months, with significantly longer OS of 27.3 months in patients who achieved a PSA decline of at least 50% versus 10.2 months for non-responders, starting from the first cycle of rechallenge. Moreover, Seifert et al. [24] reported 40.2 months from the first cycle of the initial [¹⁷⁷Lu]Lu-PSMA course, very encouraging at this level of the advanced stage of disease. However, it remains unclear whether a continuous extension of [¹⁷⁷Lu]Lu-PSMA beyond the sixth cycle leads to different overall survival outcomes compared to pausing [¹⁷⁷Lu]Lu-PSMA after the sixth cycle and performing a rechallenge upon significant disease progression.

The rechallenge results in our meta-analysis were influenced in part by the sample characteristics, which mostly included patients with good prior responses to [¹⁷⁷Lu]Lu-PSMA. However, extended treatment of [¹⁷⁷Lu]Lu-PSMA RLT beyond 6 cycles in mCRPC patients seems feasible, particularly for those without alternative treatment options [27, 28].

Furthermore, Gäble et al. [20] explored the feasibility of rechallenge with another radioligand [177Lu]Lu-rhPSMA-10.1 than the initial one ([177Lu]Lu-PSMA-I&T), showing a 30% PSA 50% decline. This concept warrants further assessment in larger patient cohorts as a possible strategy.

Patients with mCRPC frequently encounter significant barriers to receiving successive lines of therapy. Disease progression often leads to functional decline, cumulative treatment-related toxicities, and a growing burden of comorbidities, all of which can limit the feasibility of further systemic interventions. Moreover, real-world constraints such as access to specialized care, drug availability, and patient preference may further restrict treatment continuity. As a result, only a subset of patients are able to complete the full sequence of recommended therapeutic options, emphasizing the importance of optimizing early treatment decisions. In this context, the retreatment with a therapy that was initially effective is already a common approach in clinical practice, including chemotherapy for various malignancies, not just prostate cancer. However, unlike RLT, chemotherapy is typically administered in earlier stages of prostate cancer [29], reported overall survival for rechallenge chemotherapy with docetaxel and cabazitaxel ranges from 13.7 to 43.5 months [30–33].

Our meta-analysis presents limitations. One major limitation is the retrospective design of the included studies with small patient cohorts. Additionally, the studies varied in prior prostate cancer treatments, radioligand types, and administered radioactivity of [¹⁷⁷Lu]Lu-PSMA. The limited number of studies assessing treatment response, toxicity, and survival after the initial [¹⁷⁷Lu]Lu-PSMA RLT sequences further restricted the accuracy of outcome evaluation, resulting in heterogeneity of results. In addition, the therapies given between initial and rechallenge PSMA RLT series were unclear or missing in most studies, and the follow-up period was insufficient to evaluate the long-term effectiveness of the treatment and potential late-onset toxicities.

## Conclusion

Our results suggest that rechallenge with [¹⁷⁷Lu]Lu-PSMA-RLT is effective and safe in late/end-stage mCRPC patients. In addition, 225Ac-PSMA/177Lu-PSMA tandem therapy seems to be a promising approach to balance antitumor effect and tolerable side effects. However, further studies, especially prospective randomized controlled trials comparing different rechallenge strategies, are needed to explore the best options in this advanced stage of prostate cancer.

## Data Availability

The datasets generated and analyzed during the current meta-analysis are available from the corresponding author upon reasonable request.

## Abbreviations

PRISMA: Preferred Reporting Items for Systematic Reviews and Meta-Analyses
PROSPERO: International Prospective Register of Systematic Reviews
RCTs: Randomized Clinical Trials
NOS: Newcastle-Ottawa Scale
CTCAE: Common Terminology Criteria for Adverse Events
ROBINS-I: Risk Of Bias In Non-Randomized Studies– of Interventions
CI: Confidence Interval
OS: Overall Survival
PSA: Prostate-Specific Antigen
PSA-PFS: PSA Progression-Free Survival
mCRPC: Metastatic Castration-Resistant Prostate Cancer
PSMA: Prostate-Specific Membrane Antigen
RLT: Radioligand Therapy
TRT: Targeted Radionuclide Therapy
ADT: Androgen Deprivation Therapy
ARPI: Androgen Receptor Pathway Inhibitors
PARP inhibitors: Poly (ADP-ribose) Polymerase Inhibitors
177Lu: Lutetium-177
[177Lu]Lu-PSMA / 177Lu-PSMA: Lutetium-177 labeled PSMA-targeted radioligand
225Ac: Actinium-225
[225Ac]Ac-PSMA / 225Ac-PSMA: Actinium-225 labeled PSMA-targeted radioligand
[177Lu]Lu-rhPSMA-10.1: A specific variant of 177Lu-labeled PSMA agent
[177Lu]Lu-PSMA-I&T: A specific variant of 177Lu-labeled PSMA agent
VISION Trial: A clinical trial evaluating 177Lu-PSMA-617
TheraP Trial: A randomized trial comparing [177Lu]Lu-PSMA to cabazitaxel
REALITY Study: A prospective registry evaluating rechallenge with 177Lu-PSMA
